# Consanguinity and Its Sociodemographic Differentials in Bhimber District, Azad Jammu and Kashmir, Pakistan

**Published:** 2014-06

**Authors:** Nazish Jabeen, Sajid Malik

**Affiliations:** Human Genetics Program, Department of Animal Sciences, Quaid-i-Azam University, 45320 Islamabad, Pakistan

**Keywords:** Consanguinity, Cousin marriages, Inbreeding coefficient, Population, Azad Jammu and Kashmir

## Abstract

Kashmiri population in the northeast of Pakistan has strong historical, cultural and linguistic affinities with the neighbouring populations of upper Punjab and Potohar region of Pakistan. However, the study of consanguineous unions, which are customarily practised in many populations of Pakistan, revealed marked differences between the Kashmiris and other populations of northern Pakistan with respect to the distribution of marriage types and inbreeding coefficient (F). The current descriptive epidemiological study carried out in Bhimber district of Mirpur division, Azad Jammu and Kashmir, Pakistan, demonstrated that consanguineous marriages were 62% of the total marriages (F=0.0348). First-cousin unions were the predominant type of marriages and constituted 50.13% of total marital unions. The estimates of inbreeding coefficient were higher in the literate subjects, and consanguinity was witnessed to be rising with increasing literacy level. Additionally, consanguinity was observed to be associated with ethnicity, family structure, language, and marriage arrangements. Based upon these data, a distinct sociobiological structure, with increased stratification and higher genomic homozygosity, is expected for this Kashmiri population. In this communication, we present detailed distribution of the types of marital unions and the incidences of consanguinity and inbreeding coefficient (F) across various sociodemographic strata of Bhimber/Mirpuri population. The results of this study would have implication not only for other endogamous populations of Pakistan but also for the sizeable Kashmiri community immigrated to Europe.

## INTRODUCTION

Consanguineous unions (CU) are common in many countries of the Middle East, Africa, and Asia, including Pakistan (1-3). Populations practising such marriages include not only those isolated by geographical or sociocultural factors but also cultures where CU is a preferred choice (3,4). Various reasons advocated in favour of CU include economic benefits and the protection of property/land. It is also a common argument that marriage among close relatives offers advantages in terms of close family ties, compatibility of the bride with husband's family, stable relationships, and a low divorce rate (5). Another reason for CU is the ease of marriage arrangements (5,6). Various religious sects consider marriages among close kins a part of their faith. For instance, certain families in Pakistan, particularly the Syed are reluctant to marry the non-Syed (7-9).

The practice of consanguineous marriages is influenced by cultural, social, economic, religious, geographic and demographic factors (9-11). It is generally perceived that consanguinity is more prevalent among the underprivileged in the society. Hussain and Bittles observed a negative association between CU and maternal education in the Muslim population of India (12). Liascovich *et al*. recruited a large sample of non-malformed liveborn infants to estimate consanguinity in various populations of South America (13). The authors observed that low paternal education and occupation levels were positively associated with consanguinity. It was further observed that consanguineous couples more frequently lived in smaller towns and in an extended family environment (12). In a study carried out in Spain, Fuster and Colantonio witnessed that CU was associated with economic variables whereas second-cousin marriages corresponded largely to rural areas (14).

Kerkeni and colleagues studied two very different societies, i.e. Tunisia and Croatian islands and observed high consanguinity levels in both of these populations (11). They argued that there were different causes for the high prevalence of consanguinity in the two societies: in Tunisia, consanguinity was prevalent because of cultural factors while, in Croatia, it occurred because of very limited mate choice on isolated and remote island communities. It was concluded that the association between consanguinity and educational level and socioeconomic status needs to be taken into account in inbreeding studies in human populations, and the relationship will often be highly specific for each studied population and strongly dependent on the cultural context (11).

In Pakistan, several studies on consanguinity and inbreeding coefficient F (ICF) have been conducted on the populations of upper Punjab (15-17). These studies have shown that CU accounted for 38-59% of the total marriages, and first-cousin marriages were the most common type of marital unions. Generally, CU was observed to be associated with rural status, early age at marriage, and low socioeconomic and educational levels. The comparative accounts of consanguinity levels in the Pakistani populations have been presented by Shami *et al*. and Hussain and Bittles (18-19). There is, however, a paucity of knowledge for other northern populations, like Kashmiris, which not only share cultural and social traditions with upper Punjab and Potohar regions of Pakistan but also have strong linguistic and historic affinities with the later. Kashmir is strategically, politically, and culturally a very important region of Pakistan. This lack of knowledge is intriguing also because there are several published reports depicting an exceptionally high prevalence of CU in the Kashmiri families immigrated to Europe, including the UK (20). Hence, the primary aim of the present study was to explore the types of marital unions and to estimate consanguinity in people of various sociodemographic strata in Bhimber district, Azad Jammu and Kashmir, Pakistan, which represents Kashmiri population.

## MATERIALS AND METHODS

### Study population

**Figure 1.**
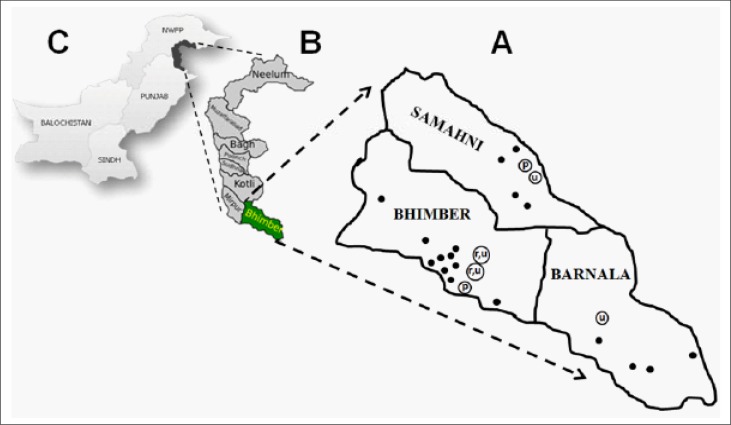
Map of Bhimber district (A) superimposed on map of Azad Jammu and Kashmir (B) and Pakistan (C). Sampling sites (n=24) in three tehsils, namely Bhimber, Samahni, and Barnala are shown with dots (rural areas) and circles (r=rural, u=urban or p=peri-urban area)

Bhimber district (32.58°N, 74.04°E) of Mirpur division is among the eight districts located at the southernmost part of Azad Jammu and Kashmir (AJK), Pakistan (Figure 1). Being a historical corridor to the north, it is regarded as a “door to Kashmir” (21). It is bordered by Sialkot and Gujrat districts in the south, Mirpur and Jhelum in the west, Kotli in the north, and Indian Kashmir in the east. On the administrative grounds, it was declared a separate district in 1995 and now comprises three tehsils─Barnala, Bhimber, and Samahni)─and 19 union councils─18 rural, 1 urban. Population of the district comprises 0.401 million individuals (2009 projections), with an annual growth rate of 2.6%. Extended family structure is common, with an average household-size of 6.7. The primary languages are Punjabi and Pahari (22,23). Pahari is mainly spoken in Samahni tehsil which is located in the northeast of Bhimber and comprises the local tribes of Kashmir. Pahari is a native language of Jammu and Kashmir whereas Punjabi is considered the language of the people who got settled here from adjoining areas in different periods. The topography ranges from plain areas to valleys and mountains. For a couple of decades, there had been a growing trend among the youths to go abroad and the remittances by Kashmiris settled in economically-developed countries form a substantial part of the district's economy (22). Other common professions are military service, manual jobs, business, and agriculture/farming. The main ethnicities in the district are: Jatt, Rajput, Gujjar, Mirza, Malik, Mughal, Bains-Rajput, Syed, and Butt (22).

### Methods

A total of 1,584 married females originating from three tehsils and 24 sampling sites of Bhimber district were randomly recruited (1,800 contacted; response rate 88%) (Figure 1) (24). A retrospective questionnaire-based study was carried out for nine months from January 2010 to September 2010. Only the married females, aged 16-70 years, who were permanent residents of Bhimber and were willing to provide complete information, were included. There were an estimated 60,109 married women of childbearing age (15-49 years) in Bhimber district (21). Hence, the proportion of the sampled marriages to the total marriages in Bhimber population was at least 0.022. Before filling of proforma, each subject was briefed about the purpose and the likely outcome of the study. After a formal informed consent from each married female, detailed information was obtained on a structured proforma on marital type, consanguinity level, and key demographic parameters (ethnicity/caste, rural/urban origin, occupation, literacy, mother tongue, family structure, etc.) of the subject and the spouse.

Sampling sites were classified as rural, peri-urban, or urban. The ‘rural’ or ‘urban’ status was established according to the census reports (22,23). A definition of ‘peri-urban’ was employed for those rural areas which were relatively developed, i.e. had acquired better health and transport facilities or had fused with the expanding urban populations. Data were also obtained on marriage arrangements: arranged, reciprocal, and self-arranged/arranged love marriages. ‘Arranged marriages’ were those in which the parents/elders of the subject played a key role in identifying the marriage partner; ‘reciprocal’ or *watta-satta* marriages involved two exchanged marriages, and ‘self-arranged or arranged love marriages’ were those in which the subject herself identified the marriage partner, and the marriage was subsequently contracted with the consent of both families.

The data were categorized by type of marriage. CU were of four types, i.e. double first cousins (DFC), first cousins (FC), first cousin once removed (FCOR), and second cousins (SC) while the non-consanguineous unions (NCU) were identified as second cousin once removed, distantly related marriages, and non-related (3). Data were analyzed separately for wife and husband (described here as subject and spouse respectively). Descriptive summaries were generated and statistical significance was calculated by employing χ^2^ and Fisher's exact test. A possible association between consanguinity and demographic variables was analyzed by comparing consanguineous with non-consanguineous marriages. The differences in the occurrences of CU within the sociodemographic variables were established with odds ratios, and the minimum number was taken as reference in each category. ICF was estimated from the weighted proportions of individual CU categories (3).

## RESULTS

### Consanguineous unions

Of the total 1,584 subjects recruited in the current study, 985 (62.18%) had CU while 599 (37.82%) had NCU, and the ICF was estimated to be 0.0348 (Table 1). FC marriages were the highest in proportion and accounted for 80.61% (n=794) of the CU (Table 2). Accordingly, DFC, FCOR, and SC marriages accounted for 1.32%, 6.80%, and 11.27% respectively. On the other hand, NCU was observed to be 37.82%. Detailed distributions of marital unions with respect to various demographic parameters of husbands and wives are given here.

**Table 1.** Consanguineous and non-consanguineous marriages and inbreeding coefficient (F) with respect to various demographic parameters of recruited subjects

Parameter	Consanguineous No. (%)	Non-consanguineous No. (%)	Total marriages No.	Odds ratio	Inbreeding coefficient (F)
Tehsil					
Bhimber	389 (63.05)	228 (36.95)	617	1.42	0.0348
Barnala	248 (54.63)	206 (45.37)	454	Reference	0.0320
Samahni	348 (67.84)	165 (32.16)	513	1.75	0.0371
Total	985 (62.18)	599 (37.82)	1,584	1.37	0.0348
	χ^2^=18.19; df.2, p=0.0001 (significant)				
Age interval (completed years)					
16-19	30 (75.00)	10 (25.00)	40	4.30	0.0441
20-29	356 (64.26)	198 (35.74)	554	2.58	0.0359
30-39	325 (63.85)	184 (36.15)	509	2.53	0.0349
40-49	177 (62.32)	107 (37.68)	284	2.37	0.0351
50-59	74 (52.48)	67 (47.52)	141	1.58	0.0311
60 and above	23 (41.07)	33 (58.93)	56	Reference	0.0229
	χ^2^=20.67; df.5, p=0.0009 (significant)				
Ethnic origin (husband)					
Jatt	454 (67.36)	220 (32.64)	674	1.97	0.0379
Rajput	131 (57.71)	96 (42.29)	227	1.30	0.0318
Gujjar	113 (51.13)	108 (48.87)	221	Reference	0.0305
Mirza	112 (58.03)	81 (41.97)	193	1.32	0.0324
Malik	35 (64.81)	19 (35.19)	54	1.76	0.0341
Mughal	33 (70.21)	14 (29.79)	47	2.25	0.0419
Bains-Rajput	35 (76.09)	11 (23.91)	46	3.04	0.0421
Syed	23 (65.71)	12 (34.29)	35	1.83	0.0326
Butt	16 (51.61)	15 (48.39)	31	1.02	0.0227
Others (n<30)	33 (58.93)	23 (41.07)	56	1.37	0.0307
	χ^2^=29.64, df.9, p=0.0005 (significant)				
Mother tongue					
Punjabi	637 (59.48)	434 (40.52)	1,071	Reference	0.0336
Pahari	348 (67.84)	165 (32.16)	513	1.44	0.0371
	χ^2^=100.6, df.1, p<0.0001 (significant)				
Location					
Rural	838 (62.03)	513 (37.97)	1,351	1.01	0.0371
Peri-urban	97 (63.82)	55 (36.18)	152	1.09	0.0346
Urban	50 (61.73)	31 (38.27)	81	Reference	0.0336
	χ^2^=0.467, df.2, p=0.7918 (non-significant)				
Family structure					
Nuclear family	386 (56.68)	295 (43.32)	681	Reference	0.0314
Extended family (all)	599 (66.33)	304 (33.67)	903	1.51	0.0370
One couple and grandparents	71 (66.98)	35 (33.02)	106	1.22	0.0308
More than one couple	10 (62.50)	6 (37.50)	16	1.00	0.0322
Extended/combined family	518 (66.33)	263 (33.67)	781	1.18	0.0379
	χ^2^=19.12, df.3, p=0.0003 (significant)				
Marriage arrangement					
Arranged traditionally	856 (60.97)	548 (39.03)	1,404	1.74	0.0335
Reciprocal/*watta-satta*	9 (47.37)	10 (52.63)	19	Reference	0.0434
Self-arranged/arranged love	120 (74.53)	41 (25.47)	161	3.25	0.0461
	χ^2^=12.17, df.2, p=0.0023 (significant)				

### Tehsil-wise distribution

A total of 617 (38.95%) subjects belonged to tehsil Bhimber, 454 (28.66%) to Barnala, and 513 (32.39%) to Samahni (Table 1). Individually in tehsils, CU ranged from 54.63% in Barnala to 67.84% in Samahni. The differences in the distribution of CU and NCU in three tehsils was statistically significant (Table 1).

### Age of subjects

The highest incidence of CU was observed in the subjects belonging to the age-range of 16-19 years (75%; ICF=0.0441), followed by 20-29 years (64.26%; ICF=0.0359) (Table 1). There was a declining trend in CU and ICF with respect to the increasing subject's age. Differences in the distribution of CU and NCU with respect to age categories were statistically significant (Table 1).

### Ethnicity/caste groups

The largest representatives belonged to Jatt community (n=674), followed by Rajput, Gujjar, and Mirza (n=227, 221, and 193 respectively) (Table 1). Among these four major ethnicities (i.e. sample-size >190), the highest rate of CU was witnessed in Jatt (67.36%), followed by Mirza (58.03%) and Rajput (57.71%) (Table 1). First-cousin (FC) marriages and inbreeding coefficient were also the highest in Jatt (55.19%; ICF=0.0379) (Table 1-2). On the other hand, in ethnicities, with sample-sizes of 30-55 individuals, the highest incidence of consanguinity was observed in Bains-Rajput (76.09%), followed by Mughal (70.21%), Syed (65.71%), and Malik (64.81%) (Table 1). Within the ethnicities, differences in the number of CU and NCU were statistically significant (χ^2^=29.64, df.9; p=0.0005) (Table 1).

### Mother tongue

With respect to the mother tongue, majority of the subjects were Punjabi-speaking (n=1071; 67.61%) while there were 513 (32.39%) subjects speaking Pahari (Table 1). The incidence of CU was higher in subjects speaking Pahari language compared to subjects speaking Punjabi (67.81% vs 59.48% respectively). First-cousin unions were 53.61% in Pahari-speaking subjects compared to 48.46% in the Punjabi-speaking individuals (Table 2).

### Rural/urban status

Majority of the subjects originated from rural areas (85.29%; n=1,351), followed by subjects belonging to peri-urban (9.6%) and urban (5.11%) areas (Table 1). The proposition of CU was slightly higher in the peri-urban sample (62.03%) compared to the rural and urban cohorts.

**Table 2.** Relative percentages of different marriage types across various demographic parameters

Parameter	Consanguineous (%)				Non-consanguineous (%)			Total no.
	DFC	FC	FCOR	SC	Second cousin once removed	Distantly related	Non-related
Tehsil								
Bhimber	0.49	50.57	4.70	7.29	3.24	15.88	17.83	617
Barnala	1.54	45.59	2.86	4.63	1.54	14.98	28.85	454
Samahni	0.58	53.61	4.87	8.77	0.39	16.96	14.81	513
Total (%)	0.82	50.13	4.23	7.01	1.83	15.97	20.01	1,584
Total (No.)	13	794	67	111	29	253	317	1,584
Age interval (completed years)								
16-19	2.50	62.50	2.50	7.50	0.00	10.00	15.00	40
20-29	0.72	51.81	5.05	6.68	1.26	13.72	20.76	554
30-39	0.39	50.69	4.91	7.86	2.16	14.54	19.45	509
40-49	0.35	52.11	3.52	6.34	2.46	15.85	19.37	284
50-59	2.84	41.84	1.42	6.38	1.42	21.99	24.11	141
60 and above	1.79	30.36	1.79	7.14	3.57	41.07	14.29	56
Ethnic origin (husband)								
Jatt	0.74	55.19	4.45	6.97	2.08	12.76	17.80	674
Rajput	0	47.58	3.08	7.05	0.88	21.15	20.26	227
Gujjar	1.81	42.99	2.71	3.62	0.45	14.03	34.39	221
Mirza	0	47.67	6.22	4.15	4.15	20.73	17.10	193
Malik	3.7	40.74	5.56	14.81	0	14.81	20.37	54
Mughal	2.13	59.57	4.26	4.26	2.13	10.64	17.02	47
Bains-Rajput	2.17	58.70	2.17	13.04	0	15.22	8.70	46
Syed	0	45.71	5.71	14.29	0	22.86	11.43	35
Butt	0	29.03	6.45	16.13	0	29.03	19.35	31
Others (n<30)	0	44.64	3.57	10.71	5.36	19.64	16.07	56
Mother tongue								
Punjabi	0.93	48.46	3.92	6.16	2.52	15.50	22.50	1,071
Pahari	0.58	53.61	4.87	8.77	0.39	16.96	14.81	513
Location								
Rural	0.81	49.52	4.44	7.25	2.07	15.77	20.13	1,351
Peri-urban	0	56.58	3.95	3.29	0.66	21.71	13.82	152
Urban	2.47	48.15	1.23	9.88	0	8.64	29.63	81
Family structure								
Nuclear family	0.59	43.47	5.14	7.49	3.08	17.77	22.47	681
Extended family (all)	1.00	55.15	3.54	6.64	0.89	14.62	18.16	903
One couple and grand-parents	0.94	50.94	3.77	11.32	3.77	15.09	14.15	106
More than one couple	0	25.00	12.50	25.00	0	0	37.50	16
Marriage arrangement								
Arranged traditionally	0.50	48.58	4.56	7.34	1.92	16.74	20.37	1,404
Reciprocal/*watta-satta*	0	42.11	5.26	0	10.53	15.79	26.32	19
Self-arranged/arranged love	3.73	64.60	1.24	4.97	0	9.32	16.15	161
Total	0.82	50.13	4.23	7.01	1.83	15.97	20.01	1,584

### Family structure/household

There were 903 (57%) respondents having extended family structures while 681 (43%) subjects belonged to nuclear families (Table 1). The subjects with CU had higher tendency of belonging to the extended families.

### Marriage arrangements

Regarding the marriage arrangements, majority of the marital unions were arranged traditionally (88.64%; n=1,404/1,584) while there were 1.20% reciprocal marriages and 10.16% self-arranged/arranged love marriages (Table 1). The highest proportion of CU was observed in self-arranged/arranged love marriage category (74.53%), followed by marriage arranged following traditional system (60.90%). The differences of the CU and NCU between these categories were statistically significant.

### Occupational status

The two main categories of husband's occupation were skilled and non-skilled. The occupational status of the husbands was further categorized into 10 classes (Table 3-4). The largest number of subjects were working abroad (n=360), followed by individuals serving in the army (n=207) and engaged in labour/manual jobs (n=202). Among the occupational groups, the highest proportion of CU was observed in individuals working as drivers (67.44%), followed by persons engaged in office-jobs (65.45%) (Table 3). ICF was calculated to be the highest in drivers, businessmen/shopkeepers, and foreign job-holders (0.0396, 0.0368, and 0.0358 respectively). The differences among CU and NCU in different occupational groups were statistically non-significant (Table 3-4).

With respect to the occupational status of wife, there were only four categories (Table 3). Majority of the women were housewives (n=1,406; 88.76%). The differences between types of marriage with respect to wife's occupation were statistically non-significant.

### Educational level

In order to check the relationship between consanguinity and education, distribution of marital unions was checked with respect to the literacy levels of the husband and wife. Majority of the husbands were literate (n=1,382; 87.25%). Consanguinity was observed to be significantly higher in the literate group compared to non-literate (64.04% vs 49.50%; p<0.0001) (Table 3). Additionally, FC unions and ICF were higher in the literate group compared to the non-literate (52.24% vs 35.64%, and 0.0362 vs 0.0253 respectively) (Table 3-4). Within the literate group, growing trends of CU and ICF were witnessed with the increasing literacy levels.

A literacy rate of 66.79% was observed in subjects/spouses (33.21% non-literate) (Table 3). Consanguinity was higher in the literate group compared to the non-literate category (65.69% vs 55.13%; p<0.0001). Additionally, the frequencies of FC marriages and ICF were also higher in the literate group (Table 3-4). There was, however, no decreasing trend in CU with respect to increasing educational levels.

## DISCUSSION

The present study confirmed that similar to other Pakistani populations, there was a very high proportion of CU in Bhimber, Kashmir (62%). The ICF in the study population (0.0348) was the highest compared to any other reported population from Pakistan. For instance, it was higher than in Lahore (0.0269), Mianchannu (0.0236), Muridke (0.0240), Sheikhupura (0.0271), Gujrat (0.0257), Jhelum (0.262), Rawalpindi (0.0286), Faisalabad (0.0293), Gujranwala (0.0323), Sahiwal (0.0295), Sialkot (0.0261), and Quetta (0.0217) (15,17,25,26). Compared to the regional cultures, the ICF in Bhimber, Kashmir, was higher than in the populations of Indonesia (0.0095); Muslims in West Bengal, India (0.0135); Muslims in Bihar, India (0.0076); Muslims in Delhi, India (0.0180); Muslims in Lucknow, India (0.0095); tribal Turkmen in Iran (0.0077); Bahrain (0.0152); Baghdad, Iraq (0.0225); and Riyadh, Saudi Arabia (0.0174) (27-34). Estimate of consanguinity in Bhimber was also higher than the Kashmiri community residing in the UK (20). However, ICF in Bhimber was lower than tribal Qashqai population of Iran (0.0392), and several Tamil populations of South India (≥0.0390) (35-36).

**Table 3.** Consanguineous and non-consanguineous marriages and inbreeding coefficient (F) with respect to occupational status and literacy of spouses

Parameter	Consanguineous No. (%)	Non-consanguineous No. (%)	Total marriages No.	Odds ratio	95% confidence interval
Occupational status of spouse (husband)					
Skilled (all)#	314 (63.05)	184 (36.95)	498	1.06	0.8476-1.3143
Non-skilled (all)$	671 (61.79)	415 (38.21)	1,086	Reference	0.8411-1.1890
Occupational categories (husband)					
Foreign/abroad job$	227 (63.06)	133 (36.94)	360	1.26	0.8575-1.8535
Military service#	126 (60.87)	81 (39.13)	207	1.15	0.7511-1.7576
Labour/manual job$	122 (60.40)	80 (39.60)	202	1.13	0.7350-1.7263
Businessmen/ shopkeeper$	104 (62.65)	62 (37.35)	166	1.24	0.7908-1.9413
Agriculture/farmer$	88 (57.52)	65 (42.48)	153	Reference	0.6355-1.5736
Driving#	58 (67.44)	28 (32.56)	86	1.53	0.8798-2.6608
Teaching#	49 (63.64)	28 (36.36)	77	1.29	0.7352-2.2725
Skilled work#	45 (61.64)	28 (38.36)	73	1.19	0.6711-2.0999
Office job#	36 (65.45)	19 (34.55)	55	1.40	0.7368-2.6584
Unemployed$	27 (61.36)	17 (38.64)	44	1.17	0.5906-2.3303
Others (n<30)$	103 (63.98)	58 (36.02)	161	1.31	0.8329-2.0658
All categories	985 (62.18)	599 (37.82)	1,584	1.21	0.8678-1.7001
	χ^2^=3.55, df.10, p=0.9655 (non-significant)				
Occupational status of subject (wife)					
Housewife	871 (61.95)	535 (38.05)	1,406	Reference	0.8588-1.1645
Cattle-work	91 (62.33)	55 (37.67)	146	1.02	0.7150-1.4445
Teaching	11 (73.33)	4 (26.67)	15	1.69	0.5351-5.3317
Others	12 (70.59)	5 (29.41)	17	1.47	0.5165-4.2077
	χ^2^=1.34, df.3, p=0.7201 (non-significant)				
Literacy level/years of education (husband)					
Non-literate (no formal schooling)	100 (49.50)	102 (50.50)	202	Reference	0.6770-1.4771
Literate (all)	885 (64.04)	497 (35.96)	1,382	1.82	1.3497-2.4442
Primary: 1-8 year(s)	320 (61.42)	201 (38.58)	521	Reference	0.7792-1.2833
Secondary: 9-12 years	471 (65.51)	248 (34.49)	719	1.19	0.9440-1.5075
Post-secondary: 13+ years	94 (66.20)	48 (33.80)	142	1.23	0.8329-1.8166
	χ^2^=15.83, df.1, p<0.0001 (significant between non-literate and literate-all); χ^2^=2.51, df.2, p=0.2848 (non-significant (within literate categories)				
Literacy level/years of education (wife)					
Non-literate (no formal schooling)	290 (55.13)	236 (44.87)	526	Reference	0.7843-1.2751
Literate (all)	695 (65.69)	363 (34.31)	1,058	1.56	1.2584-1.9292
Primary: 1-8 year(s)	413 (66.08)	212 (33.92)	625	1.05	0.8025-1.3666
Secondary: 9-12 years	253 (65.04)	136 (34.96)	389	Reference	0.7447-1.3428
Post-secondary: 13+ years	29 (65.91)	15 (34.09)	44	1.04	0.5386-2.0053
	χ^2^=16.65, df.1, p<0.0001, significant (between non-literate and literate-all); χ^2^=0.12, df.2, p=0.9435; non-significant (within literate categories)				
All categories	985 (62.18)	599 (37.82)	1,584		

#Skilled occupational group;

$Unskilled occupational group

It was further observed that the FC marriages were the most common type of unions among the CU as well as in the total marriages. Previous studies from Arab and Muslim communities in North Africa, most of west, central and south Asia, and Pakistan showed high prevalence of the FC marriages among CU (2-4,9,11,26)

The custom of marrying a close relative is deeply embedded in the cultural norms of Pakistani society in general and is also evident in the Kashmiri population (1-3). Despite the cultural preferences for marrying within blood-connected relatives, it is pertinent to identify various sociodemographic variables associated with consanguinity. The current study showed that CU was higher in certain sociodemographic strata. For instance, among the three tehsils of Bhimber district, CU and ICF were the highest in Samahni. Likewise, with respect to language, consanguinity was observed to be the highest in subjects speaking Pahari. Pahari and Punjabi languages have different origins. Pahari is mainly spoken in Samahni tehsil whereas Punjabi is more common in Bhimber and Barnala tehsils and is considered the language of tribes who migrated from upper Punjab and settled in Kashmir.

In the present study, there was a declining trend in CU and ICF with respect to the increasing age of the subjects, i.e. consanguinity was higher in the younger population compared to the older sample. This trend may indirectly be interpreted as a time-dependent increase in consanguinity in this population. It has been previously established that the prevalence of CU among Muslims in Pakistan remained stable over the four-decade assessment period of demographic study, and there was no evidence to suggest that a decline in CU is likely in the near future (1,2). A significant increase in the incidence of consanguinity over the years has been observed in Swat, Pakistan (37). The gradual increase in the incidence of CU may partly be attributed to the deteriorating law-and-order situation at the line-of-control between AJK and Indian-occupied Kashmir, which caused separation of hundreds of families and might have reduced the mate choice. To further investigate the temporal rise in consanguinity in the Kashmiri population, it would be worthwhile to record types of parental marriage, age at marriage, and duration of marriage.

**Table 4.** Relative percentages of the types of marriage with respect to occupation and literacy of spouses

Parameter	Consanguineous (%)				Non-consanguineous (%)			Total sample (No.)
	DFC	FC	FCOR	SC	Second cousin once removed	Distantly related	Non-related
Occupational status of spouse (husband)								
Skilled (all)#	0.80	50.00	4.62	7.63	2.21	16.27	18.47	498
Non-skilled (all)$	0.83	50.18	4.05	6.72	1.66	15.84	20.72	1,086
Occupational categories (husband)								
Foreign/abroad job$	0.56	52.78	3.89	5.83	1.94	15.56	19.44	360
Army service#	1.45	47.83	4.83	6.76	1.93	16.43	20.77	207
Labour/manual job$	0.5	48.02	4.95	6.93	1.49	12.87	25.25	202
Businessmen/shopkeeper$	1.2	54.22	1.81	5.42	2.41	14.46	20.48	166
Agriculture/farming$	1.31	47.06	3.27	5.88	0.65	18.95	22.88	153
Driving#	0	60.47	4.65	2.33	1.16	13.95	17.44	86
Teaching#	0	54.55	1.3	7.79	2.6	15.58	18.18	77
Skilled work#	0	43.84	8.22	9.59	2.74	21.92	13.7	73
Office job#	1.82	43.64	3.64	16.36	3.64	12.73	18.18	55
Unemployed$	0	45.45	2.27	13.64	0	22.73	15.91	44
Others (n<30)$	1.24	47.2	6.83	8.7	1.86	16.77	17.39	161
Occupational status of subject (wife)								
Housewife	0.71	49.86	4.55	6.83	2.06	16.22	19.77	1,406
Cattle-work	2.05	52.05	2.05	6.16	0	14.38	23.29	146
Teaching	0	53.33	0	20	0	0	26.67	15
Others	0	52.94	0	17.65	0	23.53	5.88	17
Literacy level/years of education (husband)								
Non-literate (no formal schooling)	0	35.64	5.45	8.42	1.49	19.31	29.7	202
Literate (all)	0.94	52.24	4.05	6.8	1.88	15.48	18.6	1,382
Primary: 1-8 year(s)	0.77	49.52	4.61	6.53	2.3	16.51	19.77	521
Secondary: 9-12 years	1.25	52.99	3.76	7.51	1.53	15.02	17.94	719
Post-secondary: 13+ years	0	58.45	3.52	4.23	2.11	14.08	17.61	142
Literacy level/years of education (wife)								
Non-literate (no formal schooling)	1.52	42.21	3.99	7.41	1.9	19.39	23.57	526
Literate (all)	0.47	54.06	4.35	6.81	1.8	14.27	18.24	1,058
Primary: 1-8 year(s)	0.48	53.76	4.96	6.88	1.28	14.24	18.4	625
Secondary: 9-12 years	0.26	56.04	3.08	5.66	2.57	13.88	18.51	389
Post-secondary: 13+years	2.27	40.91	6.82	15.91	2.27	18.18	13.64	44
All categories	0.82	50.13	4.23	7.01	1.83	15.97	20.01	1,584

#Skilled occupational group;

$Unskilled occupational group

Hussain and Bittles conducted a study in Southern Pakistan and showed that consanguinity was higher in rural communities (2). Wahab and Ahmad observed that, in Swat, northwestern Pakistan, consanguinity was higher in the sample drawn from rural areas (37). Hussain and Bittles also observed these trends in India (2). The present study corroborates findings from these studies. Additionally, CU was higher in subjects belonging to extended family structures. In a typical Pakistani rural community, an extended family structure is characterized by multiple and overlapping generations. The kindred has large sibships in each generation and majority of its members are residing in a close neighbourhood. This situation allows easy marriage arrangements between blood-connected relatives resulting in several consanguineous loops. On the other hand, the urban communities usually have multi-ethnic assemblage with small family-sizes and reduced sibships.

In the present sample, almost all of the marriages were ‘traditionally arranged’. In a traditionally-arranged union, the major marriage-related decisions are made by the couple's parents who feel the obligation to facilitate marital contracts for their children. On the other hand, ‘reciprocal marriages/*watta-satta’* which are exchanged marital unions were observed only in a small fraction. The ‘arranged love marriages’ are also convened by the parents and are usually within close relatives; in these marriages, bride/bridegroom or the couple has influenced the decision of parents or have engineered the situation almost entirely by themselves. Such marriages had been relatively infrequent but are increasing with time (38).

Literacy has been shown to be associated with consanguinity but the type of association may not always be inverse. It is generally believed that education has a decreasing effect on the frequency of CU (13,14). However, in societies where a significant change in the social system has not occurred, education does not have a decreasing effect but may have an increasing impact in contracting CU because of social pressure and political and economic imperatives (37). In the Bhimber population, literacy levels of 87.25% and 66.79% were observed for husband and wife respectively, which are remarkably higher than the average national estimates (63% and 38% respectively) (23). These data revealed that consanguinity and ICF were higher in the literate sample. Furthermore, CU was increasing with the increasing women's education, which is consistent with the observation of Wahab and Ahmad (37). The positive relationship between consanguinity and literacy may indirectly be associated with the economic status of subjects, i.e. literacy is higher in economically better-off families who prefer arranging marriages among close relatives whereas subjects from low socioeconomic strata not only have low literacy level but also tend to marry more often beyond the close kinships.

Contrasting to our findings, Kerkeni *et al*. showed that consanguinity was significantly increasing with decreasing educational level of females but not with literacy level of males (11). The authors conclude that, even in countries where consanguinity is prevalent because of cultural practices, the association with educational level and occupation status is mainly seen among women but not in men. These findings have clear implications for design and conduction of genetic epidemiological studies that investigate the effects of consanguinity on human health. For men, the nature of association strongly depends on the cultural context, with reported examples ranging from negative correlation between inbreeding and social status to a notion that more educated men were more likely to be married to cousins (39-40).

### Limitations

The present study has several potential limitations. First, it recruited only the female sample and does not present data from the male subjects. Majority of these individuals were recruited through door-to-door survey, and this sampling method could be biased towards housewives, and the data on working women who remain missing during early hours of the day may be underrepresented. Second, due to the lack of awareness about epidemiological surveys, several of the subjects were hesitant to provide complete information. Particularly, the response rate was low from couples in the non-literate community. This may be, at least partly, attributed to the deteriorating law-and-order situation in the area, a previous unpleasant experience with field workers/health visitors, or fear of misuse of personal data. However, the large sample ascertained in this study is anticipated to compensate several of these biases. Third, direct information on the economic status could not be gathered. In the rural areas, the size of agricultural land and the number of cattle are important indicators of economic status. Majority of the respondents could not provide this information accurately; hence, we have not analyzed the data on economic status. Fourth, this study does not present the association, if any, between consanguinity and fertility and health/disease status of the subjects.

Additionally, the measures of consanguinity and ICF presented here may be considered rather conservative estimates. It has been argued that marital unions are influenced by the parental marriage types (18). As reliable information on the levels of inbreeding in previous generations was not gathered (although it is known that CU had been contracted), these estimates must be regarded as minimal. Furthermore, the parental consanguinity varies greatly among countries and cultures, and the estimation of consanguinity and ICF are strongly dependent on ascertainment methods. For instance, the study of marriages through civil or religious records is limited to only certain sociocultural strata; studies based on offspring are limited to fertile matings; and the study of surnames or matrimonial distances is limited to indirect indicators. Therefore, while no approach is the ideal one, none is dispensable either, and the best knowledge about the breeding structure will result from a broad vision of that given population (13).

### Conclusions

Consanguinity and types of marriage have been studied in detail in Bhimber district of AJK, the knowledge of which would be helpful in understanding the livelihood and cultural and social aspects of this population. It is envisaged that the information gained through this study would be valuable in identifying further affinities of Bhimber/Mirpuri population with the neighbouring populations. Percentile breakdown of the Bhimber population in sociodemographic variables would be useful in understanding the dynamics of consanguinity and ICF. This study would have implication not only for rest of the Kashmiri (and other consanguineous) populations of Pakistan but also for the sizeable Kashmiri/Pakistani communities settled in Europe. For instance, several reports have highlighted an alarmingly high incidence of CU in the immigrated minorities, particularly Kashmiri-Pakistanis in the UK (9,41). The consanguineous couples are 13 times more likely than the general population to produce children with genetic disorders, and one in 10 children of first-cousin marriages in Birmingham, UK, either dies in infancy or develops a serious disability (9,41). In this context, the present study would set a baseline to quantify the genetic burden, mutational load, and population stratification in the Kashmiris that are due to consanguinity.

## ACKNOWLEDGEMENTS

The study was supported by HEC-Islamabad and URF-QAU Islamabad. We are highly indebted to the subjects and their families for voluntary participation in this study. The helpful comments of Prof. Dr. Mahmud Ahmad, Dr. SA Shami, Dr. Afsar Mian, and Dr. SM Nasim are highly acknowledged. We are also indebted to the eminent reviewers for their suggestions on the earlier versions of the manuscript.
